# Complexity of Motor Sequences and Cortical Reorganization in Parkinson's Disease: A Functional MRI Study

**DOI:** 10.1371/journal.pone.0066834

**Published:** 2013-06-25

**Authors:** Stefano Caproni, Marco Muti, Massimo Principi, Pierfausto Ottaviano, Domenico Frondizi, Giuseppe Capocchi, Piero Floridi, Aroldo Rossi, Paolo Calabresi, Nicola Tambasco

**Affiliations:** 1 Clinica Neurologica, Azienda Ospedaliera - Università di Perugia, Perugia, Italy; 2 Servizio di Fisica Sanitaria, Azienda Ospedaliera di Terni, Terni, Italy; 3 Servizio di Neuroradiologia, Azienda Ospedaliera di Terni, Terni, Italy; 4 U.O. Neurologia, Azienda Ospedaliera di Terni, Terni, Italy; 5 Servizio di Neuroradiologia, Azienda Ospedaliera di Perugia, Perugia, Italy; 6 I.R.C.C.S. – Fondazione S.Lucia – Roma, Italy; Florey Institute of Neuroscience & Mental Health, Australia

## Abstract

Motor impairment is the most relevant clinical feature in Parkinson's disease (PD). Functional imaging studies on motor impairment in PD have revealed changes in the cortical motor circuits, with particular involvement of the fronto-striatal network. The aim of this study was to assess brain activations during the performance of three different motor exercises, characterized by progressive complexity, using a functional fMRI multiple block paradigm, in PD patients and matched control subjects. Unlike from single-task comparisons, multi-task comparisons between similar exercises allowed to analyse brain areas involved in motor complexity planning and execution. Our results showed that in the single-task comparisons the involvement of primary and secondary motor areas was observed, consistent with previous findings based on similar paradigms. Most notably, in the multi-task comparisons a greater activation of supplementary motor area and posterior parietal cortex in PD patients, compared with controls, was observed. Furthermore, PD patients, compared with controls, had a lower activation of the basal ganglia and limbic structures, presumably leading to the impairment in the higher levels of motor control, including complexity planning and execution. The findings suggest that in PD patients occur both compensatory mechanisms and loss of efficiency and provide further insight into the pathophysiological role of distinct cortical and subcortical areas in motor dysfunction.

## Introduction

The study of movement complexity certainly represents one of the most difficult challenges for researchers. Clinical observations in patients with apraxia and motor incoordination and experimental studies using invasive techniques have confirmed the role of specific cortical areas during the execution of a fixed movement [1]–[5]. Default mode, motor and cognitive networks governing motor activities encode motor gestures in their relevant qualitative characteristics such as direction, strength, rate and frequency [6]–[10]. Higher levels of control also contribute to spatial and temporal coordination of single movements enabling to execute a sequence of motor gestures which is characterized by: (i) the number of muscles that are involved, (ii) the number of single movements that constitute the sequence, and (iii) single gesture temporal connections. All these components converge in determining movement complexity [11]–[14].

Over the last decades structural and functional neuroimaging techniques have enabled to investigate motor execution in living human brain in physiological non invasive conditions, also allowing to verify the impairment of cortical activity in several motor disorders. Both by positron-emission tomography (PET) and functional magnetic resonance imaging (fMRI) the cortical activation involved in fixed motor gestures has been studied with good spatial and temporal resolution. fMRI offers greater benefits due to its major spatial resolution and non-invasiveness, which favor repeatability and accuracy of results. Cerebral and cerebellar cortices, basal ganglia and limbic system have been extensively studied using fMRI. The application of fMRI to the study of brain motor organization verified the role of primary motor areas (M1) [15], [16], secondary motor areas [17], [18], and basal ganglia [19] in motor planning and execution.

These neuroradiological techniques were therefore applied to verify the impairment of cortical activities in several motor disorders. Parkinson's disease (PD) is one of the most studied movement disorders, predominantly by fMRI. In the last fifteen years, many functional studies have pointed out the impairment in activation of primary and secondary motor areas, compared to control subjects. The over-activation of bilateral M1, premotor cortex and supplementary motor area (SMA) has been observed through different tasks in basal conditions; these alterations are modified by either training or pharmacological treatment [20]–[26]. Moreover, the dopamine defect in PD leads to the alteration of frontostriatal and corticocortical connectivity [23], [27]–[30]. In fact, previous studies have detected the subsequent compensatory function supplied by other brain regions such as prefrontal cortex, cingulate cortex, precuneus and ipsilateral cerebellar cortex [20], [23], [24], [26], [28]. All these findings derive from studies focused on a single task designed to investigate a specific movement in PD patients such as finger tapping, simple motor sequences or wrist flexion-extension. However, a comprehensive overview about the mechanisms of impairment in motor complexity in PD is still missing because of the variability of the single findings. A motor paradigm comparing exercises having gradually increasing complexity is required to investigate motor complexity planning and execution. For these reasons, we selected three consecutive motor tasks, characterised by a different number of single movements and a temporal sequence of single gestures, and defined a fMRI multiple block paradigm. This specific multi-task comparison represents an experimental approach to analyse the patterns of cortical activation involved in the higher levels of motor control such as motor coordination and motor complexity planning. Thus, the aim of the present study is to assess brain activations during the performance of these different motor tasks, characterized by progressive complexity, in both PD patients and matched controls.

## Materials and Methods

### Subjects

Eleven right-handed PD patients (8 males, 3 females; mean age: 65 years, range: 59–75), mean disease duration 3.8 ± standard deviation 1.5 years (mean UPDRS off-state: 20±4.5; mean Hoehn and Yahr scale: 2, range 1–3, all treated with levodopa (mean dose: 500 ± 100 mg daily) were recruited. Eleven healthy right-handed age and sex-matched subjects served as controls (Table 1). PD clinical diagnosis was defined in accordance to the United Kingdom Parkinson's Disease Brain Bank [31]. None of the control subjects had history of past or present neurological, cardiovascular or psychiatric diseases. All participants gave their informed consent to the study. The local ethics committee approved the study.

**Table 1:** Clinical characteristics of patients and control subjects.

PD patients	Sex	Age	Disease duration	Hohen and Yahr	UPDRS in off-state
1	m	59	3	1,5	14
2	m	75	3	2	19
3	m	58	2	1	19
4	m	64	3	2	19
5	m	64	5	2	14
6	m	65	5	2,5	25
7	m	67	4	2	24
8	m	69	5	3	30
9	f	60	5	2	18
10	f	65	2	1	8
11	f	69	5	3	30
Mean		65	3,8	2	20
Controls					
1	f	60			
2	m	76			
3	f	56			
4	m	68			
5	m	63			
6	m	63			
7	m	66			
8	m	71			
9	m	59			
10	m	64			
11	f	70			
Mean		65,1			

### Ethics Statement

All participants gave their written informed consent to the study. The local ethics committee (Umbria CEAS) approved the study. The clinical investigation must have been conducted according to the principles expressed in the Declaration of Helsinki

### Motor task

All subjects performed a motor task, consisting of three exercises of increasing difficulty with the right hand. Subjects were asked to press the five keys of an in-house made keyboard, connected to the magnetic resonance console to enable the observers to monitor the whole test. The five keys were numbered from 1 to 5 and corresponded to the five fingers of the hand (thumb – finger – middle – annular – little finger), whereas, the experimental task consisted of 3 exercises:

- FINGER: repetitive alternating tapping of key 2;
- SIMPLE SCALE: 1 – 2 – 3 – 4 – 5 sequence tapping (repeated);
- COMPLEX SCALE: 1 – 3 – 5 – 2 – 4 sequence tapping (repeated).

Subjects were instructed to practice the task for about 10 minutes right before the fMRI scanning, to avoid over-training and mnemonic learning of the motor sequences. All exams were performed at the same hour (3 or 4 p.m., in off-state). In order to avoid other cognitive processes (e.g. attention on an external pacing, or the sensorial monitoring of movement frequency) to mask the processes involved in complex motor planning and execution, subjects were asked to execute the motor sequences in a self-paced way, in order to best perform the task. Data acquisition for the entire task was obtained during a single magnetic resonance scan.

### fMRI data acquisition

In this study a 1.5 T Philips scanner was used, equipped with whole-brain single-shot 3D Blood Oxygen Level Dependent echoplanar imaging (EPI) hardware. Subjects laid in the scanner and could read the instructions displayed on a white panel placed in the front of the scanner. Head pads and a firm chin strap immobilised head flexion–extension. Thirty-four axial slices of 4 mm thickness, parallel to the intercommisural plane (from z = −50 mm to z = +80 mm), were collected using an EPI gradient echo sequence, echo time = 50 ms; repetition time (TR) = 3000 ms; flip angle = 90°; field of view = 230 mm; voxel size = 3.59×3.59×4 mm^3^; matrix = 64×64. T1-weighted images were also acquired.

Data acquisition was organized in an epoch-related design. Acquisition time was divided into rest periods followed by active periods. Each period consisted of 7 EPI acquisitions of 3000 ms (TR) each, 21 s in total. The 3 exercises were performed for 4 periods, with a total of 12 rest and 12 active periods, divided into 168 volumes (4 repetitions of REST-FINGER, then 4 repetitions of REST-SIMPLE SCALE, then 4 repetitions of REST-COMPLEX SCALE). Tasks lasted 504 s, corresponding to 8 min and 24 s.

Active and rest periods were indicated through the projection of a corresponding command (FINGER, SIMPLE SCALE, COMPLEX SCALE, REST) displayed on the panel, during the first 3 seconds (1 dynamic), followed by a white screen on the panel, to minimise the visual afferent effect on cerebral activation.

### Data analysis

fMRI data were analyzed using SPM v2 and v5 (Statistical Parametric Mapping, Wellcome Department of Cognitive Neurology, London, UK) [32]. The functional images were co-registered and realigned to the first volume to correct for head translation or rotation during the scanning and to avoid incorrect spatial coordinates of activated voxels, as well as normalised using a standard voxel size 2×2×2 mm^3^ to the stereotaxic space of Talairach and Tournoux [33] using the three-dimensional volume [34]. The images were also spatially smoothed with a Gaussian kernel of 8 mm full-width half maximum and temporally smoothed with a Gaussian kernel (FWHM = 8 s) [35].

Statistical analysis of the activation obtained during task performance was based upon an epoch-related experimental design. The data obtained were modeled with a hemodynamic response function, with impulsive local flux variation. The sum of hemodynamic variations during an active period allowed for the calculation of mean cortical activation during an exercise performance. Thus, the whole brain mean activation signal corresponding to a motor exercise (FINGER, SIMPLE SCALE, COMPLEX SCALE) was compared to the rest status (REST), performing a first-level fixed-effect analysis having a cluster threshold of 10 voxels with *p*<0.001. Furthermore, the signals of two motor exercises was also compared (SIMPLE SCALE>FINGER; COMPLEX SCALE>FINGER; COMPLEX SALE>SIMPLE SCALE). A General Linear Model, y = (β/βerr)*x + c [36] was applied to obtain, for each cortical area and subcortical region activation, the corresponding T-score. This score reflects its activation size (cluster of voxels, k) and also the coordinates of the local maxima in the stereotaxic space of Talairach and Tournoux. This process was applied to all subjects.

Group data were obtained through a random-effect second-level analysis using the SPM5 software package and were used to calculate a between-group analysis. Using this experimental design it was also performed a mixed-effect analysis which compared between-group differences resulting from the contrast between the execution of two exercises (e.g., PD {COMPLEX SCALE>FINGER} > control {COMPLEX SCALE > FINGER}) through a two way t-test. The second-level analysis provided results on over-activations of whole brain cortical areas and subcortical regions, with intensity measured by a F-score.

In summary, three single-task analysis were performed:

- PD patients (FINGER > REST) vs Controls (FINGER > REST);
- PD patients (SIMPLE SCALE > REST) vs Controls (SIMPLE SCALE > REST);
- PD patients (COMPLEX SCALE > REST) vs Controls (COMPLEX SCALE > REST).

Furthermore, two multi-task analysis were operated:

- PD patients (SIMPLE SCALE > FINGER) vs Controls (SIMPLE SCALE > FINGER);
- PD patients (COMPLEX SCALE > SIMPLE SCALE) vs Controls (COMPLEX SCALE > SIMPLE SCALE).

## Results

### Behavioural results

All subjects correctly carried out the motor task. Although no fixed paced frequency was given by the investigator, homogenous values were obtained for all subjects for each group, and no statistical differences between groups were observed at t-test (Table 2). None of the subjects performed any visible movements other than those required by the task.

**Table 2:** Mean tapping frequency of each task in controls and patients.

Controls	Mean frequency ± SD (Hz)	Patients	Mean frequency ± SD (Hz)
FINGER	1.2 ± 0.15	FINGER	1.0 ± 0.20
SIMPLE SCALE	1.1 ± 0.22	SIMPLE SCALE	0.9 ± 0.24
COMPLEX SCALE	1.0 ± 0.25	COMPLEX SCALE	0.8 ± 0.30

Controls and patients did not differ in mean tapping frequency at t-test (p>0.05).

### fMRI: single-task analysis

**Figure 1:**
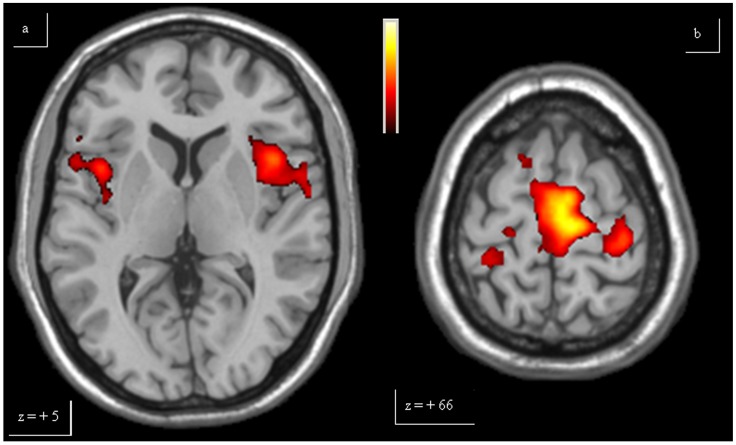
Single-task analysis Finger. The over-activations of bilateral insula (a), bilateral primary motor area and caudal supplementary motor area (b) observed in patients, compared to controls, for Finger, are shown. Colours bar range for F-score: 2 to 8.09.

**Figure 2:**
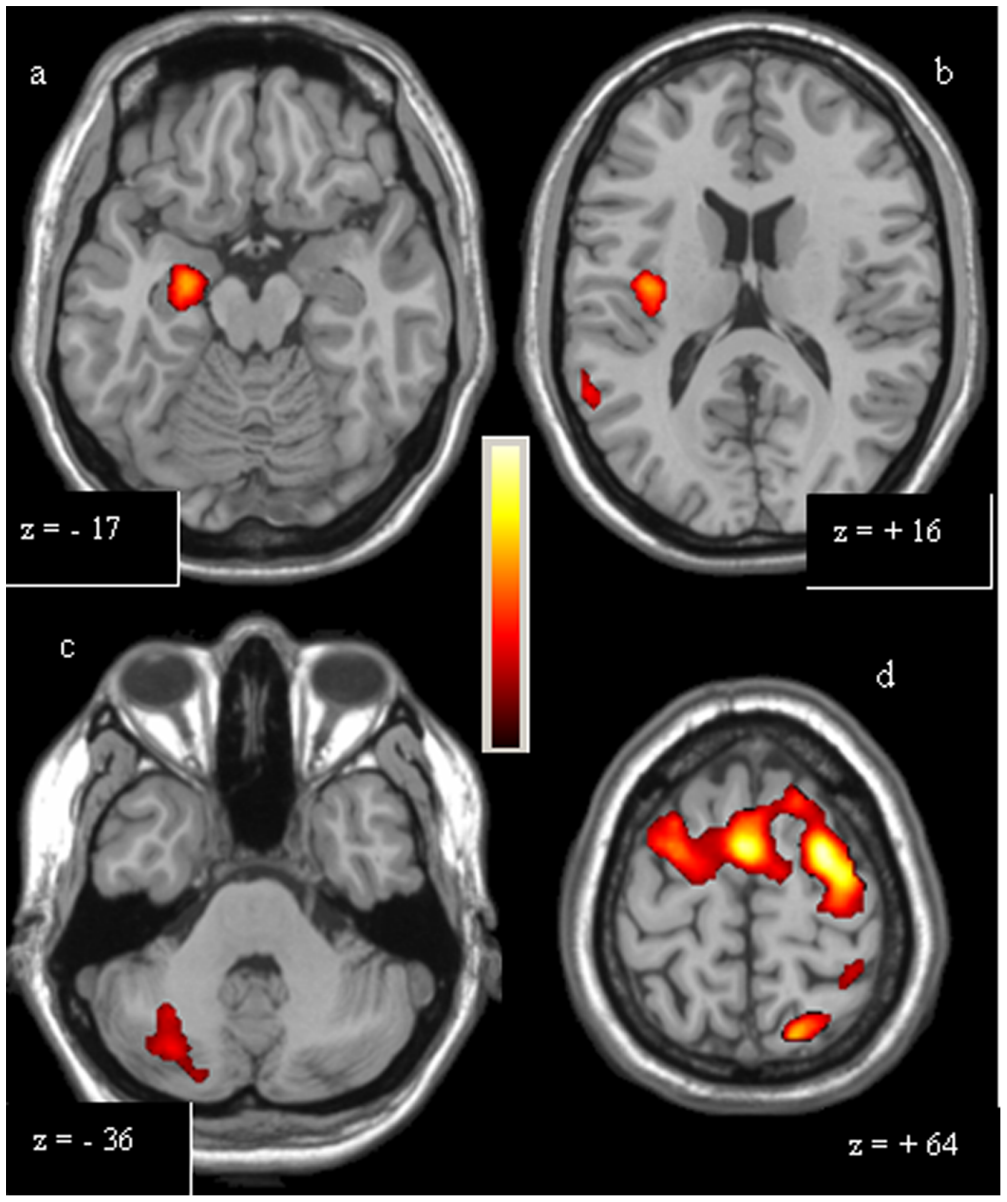
Single-task analysis Simple Scale. The over-activations of right hippocampus (a) and right insula (b) observed in controls, compared to patients, and of right cerebellum (c), left primary motor area, right dorsolateral prefrontal cortex and rostral supplementary motor area (d) observed in patients, compared to controls, for Simple Scale, are shown. Colours bar range for F-score: 2 to 8,63.

**Figure 3:**
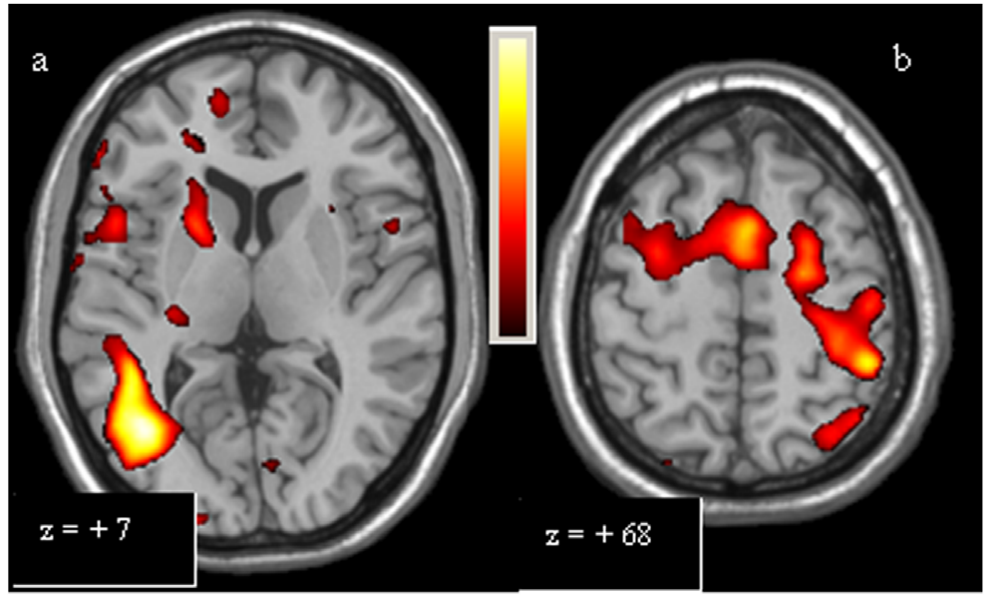
Single-task analysis Complex Scale, PD Patients > Controls. The over-activations of right putamen, right inferior temporal cortex (a), left primary motor area, right dorsolateral prefrontal cortex, left posterior parietal cortex and rostral supplementary motor area (b) observed in patients, compared to controls, for Complex Scale, are shown. Colours bar range for F-score: 2 to 9,47.

**Figure 4:**
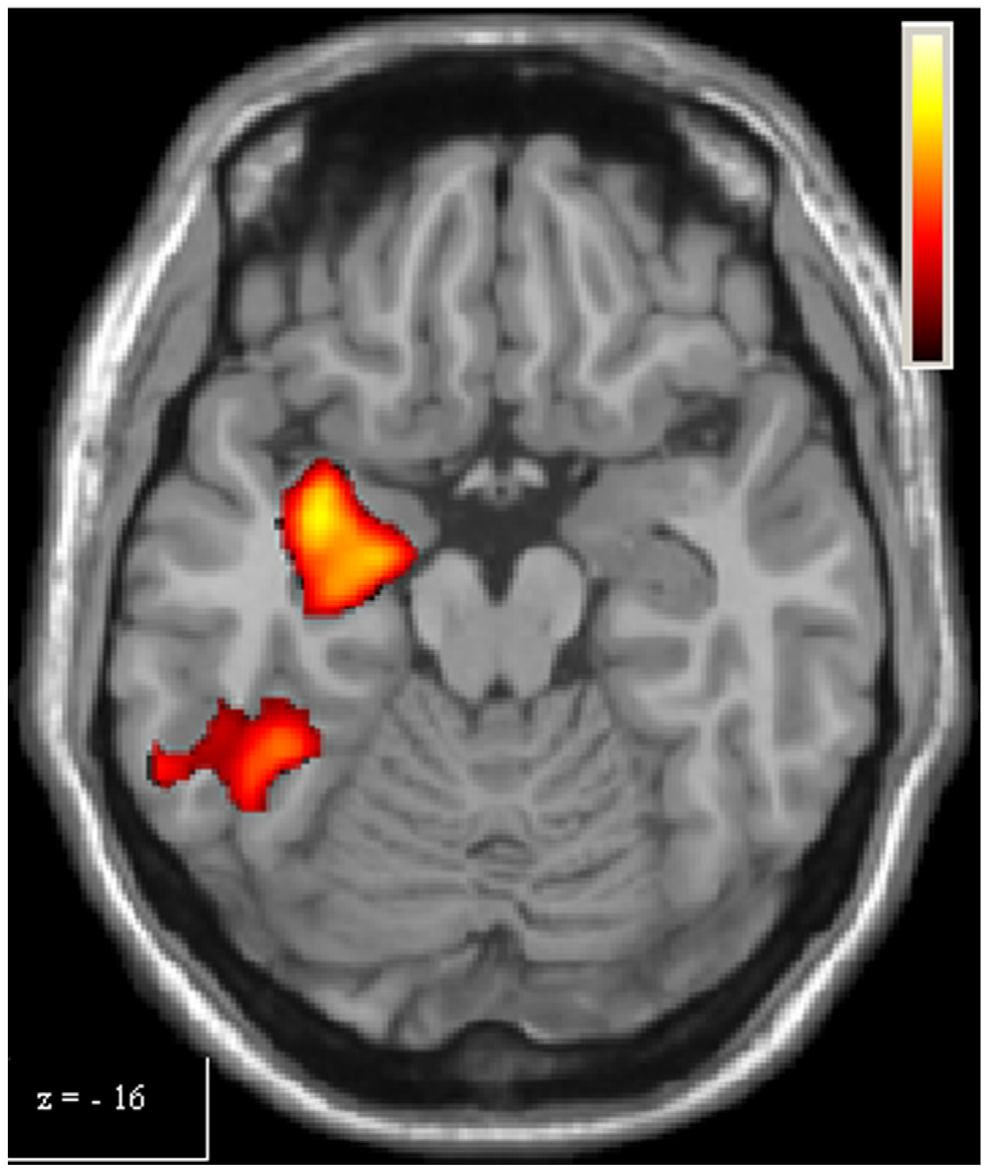
Single-task analysis Complex Scale, Controls > PD Patients. The over-activation of right hippocampus observed in controls, compared to patients, for Complex Scale, is shown. Colours bar range for F-score: 2 to 9,47.

Compared to controls, for FINGER PD patients showed an over-activation of caudal SMA (cSMA, also known as SMA-proper), bilateral (M1) and insula, anterior cingulated cortex (ACC) and right posterior parietal cortex (PPC), defined as the union of Broadman areas (BA) 7 and 40 (Figure 1, Table 3). Controls did not show over-activations compared to PD patient for FINGER. During the execution of SIMPLE SCALE, in the PD group, compared with the controls, the following over-activations were observed: left M1, rostral supplementary motor area (rSMA, also known as pre-SMA), right dorso-lateral prefrontal cortex (DLPFC), bilateral PPC, right inferior temporal cortex (ITC, BA 37), ACC, left insula and right cerebellum. For the same task, controls showed greater activations for the posterior cingulated cortex (PCC), left pallidum, right hippocampus and right middle temporal cortex, compared with PD patients (Figure 2, Table 3). Finally, during COMPLEX SCALE, PD patients had similar results for SIMPLE SCALE compared to controls, with also the over-activation of the right striatum (both putamen and caudate) (Figure 3). Conversely, compared to PD patients, controls had over-activations of PCC, right hippocampus and the left striatum (putamen) (Figure 4, Table 3).

**Table 3:** Single-task analysis results.

Task	Comparison	Area	Coordinates (mm)			T-score	cluster size
			x	y	z		
Finger	PD > Controls						
		cSMA	6	−10	60	8,09	644
		L M1	18	−20	72	6,43	401
		R M1	−20	−30	72	4,95	136
		L Insula	42	14	6	5,24	339
		R Insula	40	8	−2	5,33	213
		R PPC	−54	−44	44	5,4	113
		ACC	8	46	34	4,5	111
Simple Scale	PD > Controls						
		L M1	44	−16	58	6,31	147
		rSMA	0	2	58	8,63	2259
		R DLPFC	−42	10	36	5,89	837
		L BA 7	18	−66	62	7,07	518
		L PPC	44	−40	56	6,92	498
		R PPC	−48	−46	46	6,52	341
		R ITC	−52	−68	2	6,68	115
		R Cerebellum	−34	−76	−50	5,72	537
		R Insula	−46	14	−8	5	205
		ACC	−10	20	28	6,19	128
	Controls > PD						
		PCC	18	−40	54	7.13	1760
		L Pallidum	−12	−10	−6	5,68	217
		R Insula	34	−14	−6	5,62	245
		R Hippocampus	24	−8	−12	5,37	332
		R middle temporal cortex	58	−60	22	4,76	249
Complex Scale	PD > Controls						
		L M1	34	−28	52	6,29	109
		rSMA	2	6	54	7,33	1576
		R DLPFC	−58	8	24	5,88	321
		R Putamen	−20	10	6	6,73	144
		R Caudate	−18	14	4	5,12	172
		R PPC	−24	−76	46	6,42	419
		L PPC	50	−40	52	8,72	578
		R ITC	−48	−66	2	9,47	140
		R Cerebellum	−6	−52	−12	5,13	216
	Controls > PD						
		L Putamen	−26	−16	10	5,37	68
		Precuneus	18	−40	44	7,22	181
		R Hippocampus	22	−8	−12	7,57	340
		PCC	14	−16	46	8,31	980

Results of whole brain fMRI between group analysis are reported. For each region with significant differences Talairach coordinates and T-score of the local maxima are reported (*p*<0.001). ACC: anterior cingulated cortex; DLPFC: dorso-lateral prefrontal cortex; ITC: inferior temporal cortex; L:left; M1: primary motor area; PPC: posterior parietal cortex; PD: Parkinson's disease; R: right; cSMA: caudal supplementary motor area; rSMA: rostral supplementary motor area.

### fMRI: multi-task analysis

**Figure 5:**
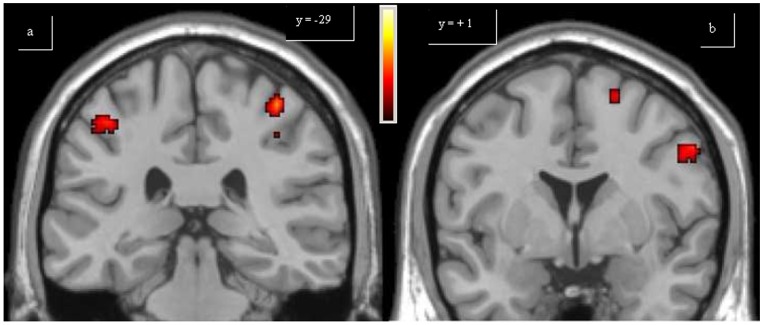
Multi-task analysis Simple Scale vs Finger. The over-activations of bilateral posterior parietal cortex (a), left primary motor area and left rostral supplementary motor area (b) observed in patients, compared to controls, for the second level analysis (Simple Scale vs Finger), are shown. Colours bar range for F-score: 2 to 27,55.

**Figure 6:**
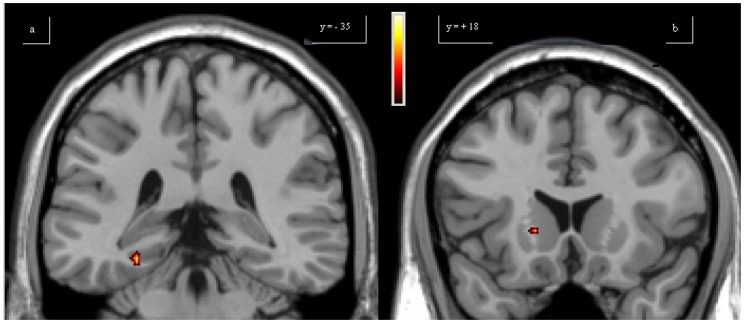
Multi-task analysis Complex Scale vs Simple Scale. The over-activations of right parahippocampal (a) cortex and caudate (b) observed in controls, compared to patients, for the second level analysis (Complex Scale vs Simple Scale), are shown. Colours bar range for F-score: 2 to 18,14.

Data obtained from second-level multi-task analysis permitted a between-group comparison on two different tasks. In SIMPLE SCALE > FINGER comparison, PD patients had greater activations of the left M1, rSMA, right cerebellum and bilateral PPC compared with controls (Figure 5). In COMPLEX SCALE > SIMPLE SCALE comparison, in PD patients higher activations of the left PPC and right cerebellum were observed compared with controls. For the same comparison, controls had greater activations of the right striatum and para-hippocampal cortex compared with PD patients (Figure 6, Table 4).

**Table 4:** Multi-task analysis results.

Task	Comparison	Area	Coordinates (mm)			F-score	cluster size
			x	y	z		
Simple Scale > Finger							
	PD > Controls						
		R cerebellum	−28	−48	−32	15,49	201
		L M1	32	−46	62	27,55	122
		rSMA	18	2	62	16,34	769
		L PPC	44	−32	50	14,47	541
		R PPC	−44	−44	56	16,32	485
Complex Scale > Simple Scale							
	PD > Controls						
		R PPC	42	−60	14	16,28	399
		R cerebellum	−32	−60	−22	15,41	185
	Controls > PD						
		R caudate	40	−6	28	14,89	60
		R para-hippocampal cortex	−28	−36	−14	18,14	75

Results of whole brain fMRI between group analysis are reported. For each region with significant differences Talairach coordinates and F-score of the local maxima are reported (*p*<0.001). L:left; M1: primary motor area; PPC: posterior parietal cortex; PD: Parkinson's disease; R: right; rSMA: rostral supplementary motor area.

## Discussion

The task used in the present study allowed for the analysis of the cortical activations involved in motor coordination along with those needed for motor complexity planning and execution, through the comparison of exercises having gradually increasing complexity.

The greater activation of bilateral M1 during the performance of FINGER observed in PD patients, compared with controls, can be interpreted, according to the literature, as a loss of selectivity of cortical activation [24]–[26]. In the same comparison, the over-activation of bilateral insula in PD patients could be considered as sign of a greater effort in task execution. In fact, a role of the insula in decision making contexts including task difficulty has been suggested [37]. Thus, our data may indicate a compensatory activation of the insula in order to optimize motor execution. The over-activation of the insula in PD patients was not observed for COMPLEX SCALE, possibly due to the complexity of the task that requires a similar effort in healthy subjects. PPC was also over-activated in PD patients compared to controls for FINGER, and this can be explained as a well-known compensatory cortical pathway of the striatal-frontal loop, which improves the motor execution through the continuous control of sensory inputs [20], [21], [23], [29], [38]–[40]. For the same task, the over-activation of ACC could be considered part of the above mentioned compensatory cortical pathway, with a specific role in temporal control of motor performance [20]. The final important result for FINGER was the greater activation of cSMA in PD patients, suggesting a greater effort in carrying out motor execution in order to reach the same accuracy of healthy subjects [20]–[22], [27].

Results from SIMPLE SCALE, as discussed for FINGER, consisted in the over-activation of left M1, bilateral PPC, left insula and ACC in PD patients than in controls. Moreover, the greater activation of right cerebellum in PD patients supports the hypothesis of the negative correlation between the activation of the ipsilateral cerebellum and controlateral striatum probably representing a compensatory mechanism, particularly for movement timing [41].

Furthermore, a greater activation of right DLPFC was observed in PD patients, compared to controls. It has been hypothesised that this area has an important role in early performance, monitoring and learning of a novel movement [23]. Thus, this greater activation might be considered compensatory to the difficulty in training of PD patients, through a continuous motor control that determines a potential “self-induced” cue, focusing attention on motor performance [22], [29], [40].

Finally, for SIMPLE SCALE an over-activation of rSMA was observed in PD patients compared to controls. This result seems to be in contrast with previous data in literature [20]–[22], [41], [42], which observed in off-state patients a reduced activation of rSMA. This finding can be explained by analysing the experimental designs of these previous studies. Firstly, some data were obtained from either naïve PD patients [22] or clinically advanced patients [20], [30]. Thus, it can be postulated that naïve patients could not have developed cortical compensatory re-organization, which can be considered inefficient in advanced PD patients. Secondly, some authors have designed motor tasks characterised by longer or more complex sequences of movements [23], [42]. This experimental design determined a difference in behavioral results between patients and controls and in this context, the hypo-activation of rSMA can be linked to the efficiency of motor execution. Third, some authors have utilized simple but externally paced sequences of movements [21], [41]; these could have determined inefficient balances between the mesial striato-frontal loop and the lateral parietal-prefrontal loop with the prevalence of the latter, causing the hypo-activation of rSMA. On the other hand, regarding SIMPLE SCALE, a hypo-activation of the left pallidum, posterior cingulate cortex (PCC) and right hippocampus was observed in PD patients. In fact, reduced activation of the left pallidum is clearly linked to the classic pathophysiological model of basal ganglia dysfunction in PD. PCC hypo-activation confirms recent evidence concerning the impairment of the default mode network in PD, resulting in a reduction in PPC activation and the projection to frontal areas. This impairment can lead to an imbalance of external interference during the execution of automatic movements [43]. The hypo-activation of the right hippocampus is a relevant finding as, to our knowledge, it was observed for the first time in an fMRI study utilizing a motor task. Few authors have investigated on the role of the hippocampus in the execution of a cognitive task applied to PD patients. The hippocampus along with the connected mesial temporal cortex are known to be crucial for declarative knowledge of movement sequences [44]. In a previous PET experience in which PD patients were studied while performing a Tower Of London problem, a greater activation of the right hippocampus was observed in PD; this finding was interpreted as a compensation for striatal impairment [45]. In contrast, our findings are consistent with recent fMRI experiences that focused on the contribution of the hippocampus in the behavioral learning process in the early motor sequence acquisition stage [46]. In fact, the hippocampus seems to have a specific role in the “accuracy” of movement (explicit and rapidly learned), in association to putamen [47]; our data could therefore be interpreted as an impairment of sub-cortical declarative functions linked to the basal ganglia involvement in PD.

Results from COMPLEX SCALE are in part similar to those from SIMPLE SCALE. In particular, the greater activation observed in PD patients are almost identical (with slight differences among T-scores and cluster sizes). PD patients also showed hypo-activations in PCC, right hippocampus, left putamen, and precuneus. The finding regarding the putamen can be easily interpreted on the pathophysiological basis of PD [48], and confirms the left hemispheric dominance of the basal ganglia observed in healthy subjects [49]. The relative hypo-activation of the precuneus in PD patients could be explained by the impairment of the default mode network [43] further supporting the hypothesis of a dysfunction in the control of automatic movements in the presence of external interference.

In the first multi-task analysis PD patients had a greater activation of the cortical areas belonging to compensatory pathways (parietal-frontal and cerebellar-frontal) and this resulted in the involvement of the left M1. In PD patients the lack of selectivity in the striatal-frontal pathway can also be responsible for rigidity and impairment in handling, which are both typically observed in PD patients [41]. The over-activation of the left M1 is due to the hyperactivity of rSMA, which is involved in motor planning, including the temporal coordination of single movements [50], [51].

In the final comparison of this study, over-activations of the left PPC and right cerebellum were observed in PD patients. For the compensatory role of these areas in PD patients is important to consider their lateralization. In fact, while we have observed the involvement of the right cerebellum in all the above comparisons, the over-activation of the PPC was usually bilateral. At this regard, we need to stress that all the subjects in the study were right-handed. Thus, the dominant hemispheric PPC was over-activated in the last comparison, in agreement with previous studies [52]. The relative defect in the right head of the caudate activation in PD patients, according to previous observation in literature [25], can therefore provide greater insight into their clinical features. In fact, while the putamen has shown to be principally involved in motor functions, the caudate nucleus, particularly its head, is thought to play a cognitive role [53]. Furthermore, the relative hypo-activation of the right hippocampus indicates the defect in complexity encoding in PD patients, connected to basal ganglia involvement [47].

## Conclusions

The design used in this study allowed to analysis the cortical activations involved in pivotal aspects of planning and execution of movement. Our findings contribute to the understanding of PD pathophysiology, revealing the role of the basal ganglia and limbic structures defect leading to the impairment in motor complexity planning and execution.
